# Percutaneous Punctured Transcatheter Device Closure of Residual Shunt after Ventricular Septal Defect Repair

**DOI:** 10.1155/2016/8124731

**Published:** 2016-05-17

**Authors:** Xuming Mo, Jirong Qi, Weisong Zuo

**Affiliations:** Department of Cardiothoracic Surgery, the Affiliated Children's Hospital of Nanjing Medical University, Nanjing, Jiangsu 210000, China

## Abstract

Ventricular septal defects (VSDs) are estimated to account for 20 to 30% of all congenital heart defects (CHDs). Although a residual shunt is the most common complication of VSD surgery, a second operation that applies the surgical repair method is very difficult because it can increase the possibility of uncontrolled bleeding and the severity of tissue adhesion. Here, we present the first case of percutaneous punctured transcatheter device closure of a residual shunt after VSD repair as a novel method to further develop for the treatment of children with congenital heart disease.

## 1. Introduction

Congenital heart defects (CHDs) are the most common type of major human birth malformation, affecting approximately 8 per 1,000 live births. Ventricular septal defects (VSDs) are estimated to account for 20 to 30% of all cases of congenital heart disease [1]. The first VSD repair was performed by Lillehei and associates in 1954 [2]. Although a residual shunt is the most common complication of VSD surgery, a second operation that applies the surgical repair method is very difficult and can increase the possibility of uncontrolled bleeding and the severity of tissue adhesion [3]. Here, we present a new avenue for the treatment of a postoperative residual shunt in VSD. We also describe the technical considerations required to perform percutaneous punctured transcatheter device closure of the residual shunt after VSD repair, and we discuss the advantages for employing these unconventional approaches.

## 2. Surgical Technique

### 2.1. General Preparation

In March 2015, a 4-year-old female patient was admitted with a residual shunt after VSD repair. She was diagnosed with congenital VSD and pulmonary hypertension when she was born. We had previously performed a thoracotomy on this patient to repair the VSD in September 2010. During long-term follow-up after surgery, an ultrasonic cardiogram (UCG) showed a 5.7 mm diameter residual shunt under the patch and mild tricuspid regurgitation, with pulmonary arterial pressure (PAP) of 27 mmHg and pulmonary circulatory blood volume/systemic circulation volume (*Q*_*p*_/*Q*_*s*_) of 1.45. Electrocardiography (ECG) revealed left atrial rhythms and an incomplete right bundle branch block. On March 5, we performed percutaneous punctured transcatheter device closure of the VSD.

In the horizontal position, transesophageal echocardiographic (TEE) assessment of the VSD was performed following the induction of general anaesthesia. TEE was utilized with every procedure to guide the conveyor system and to assess ventricular function and device interventions.

### 2.2. Operation

**Figure 1:**
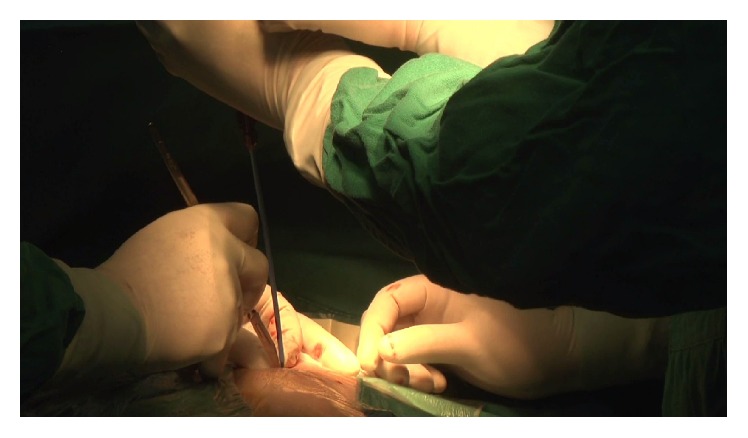
In the 4th intercostal space of the left sternal border, puncture directly through the right ventricular surface into the right ventricle with an 18 G needle, and then pass the guide wire into the left ventricle through the sheath.

**Figure 2:**
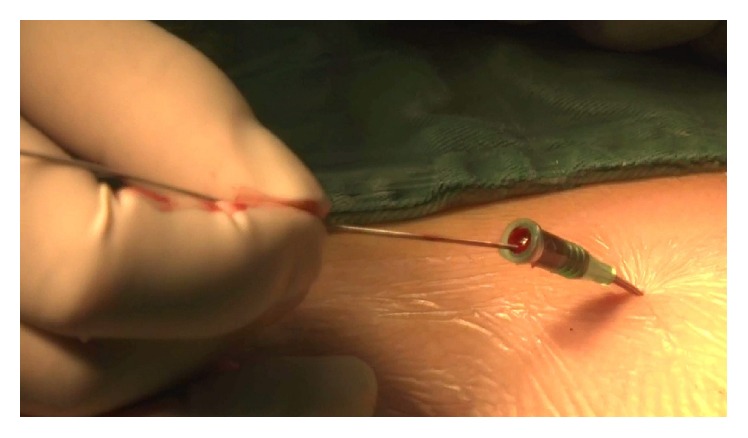
Extraction of the guide wire after insertion of a conveyor tube.

**Figure 3:**
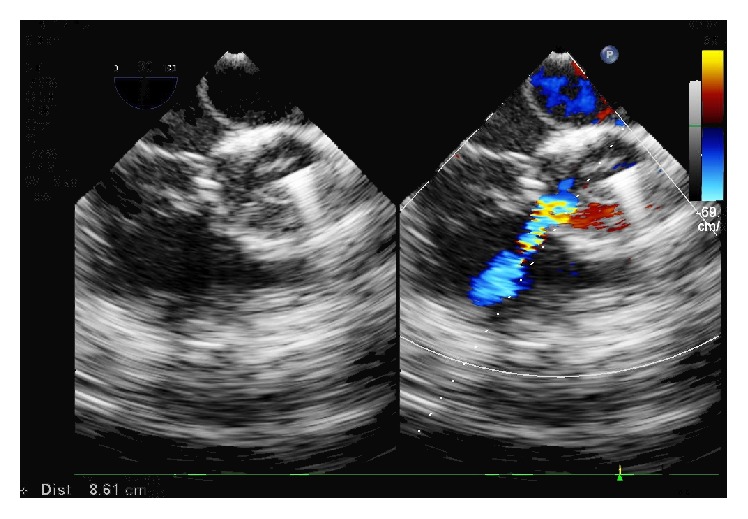
After releasing the closure device, TEE showed no shunt and no effect on the valve.

Guided by TEE, we punctured directly through the right ventricular surface into the right ventricle with an 18 G needle in the 4th intercostal space of the left sternal border, and then we inserted the guide wire into the left ventricle through the sheath (Figure 1). Next, we extracted the guide wire after inserting a conveyor tube. Subsequently, an 8 mm single-rivet double-plate closure device (WTSQFDQ-II 08, Shanghai Shape Memory Alloy, Ltd., China) was delivered via the conveyor tube (Figure 2). First, we released the left ventricular surface of the closure device, and then we released the right surface after moderate traction. TEE showed no shunt and no effect on the valve (Figure 3). Then, we retracted the conveyor and conducted compression haemostasis on the puncture site. A complete TEE examination was performed and revealed no significant pericardial effusion. The patient was transferred to the intensive care unit. UCG postoperatively showed the VSD closure device on site, with no pericardial effusion and no obvious morphological abnormalities of the valve. The patient has recovered very well.

## 3. Discussion

Surgical repair has been considered the gold standard for the treatment of most VSDs because it allows for direct visual access to the defect. Open-heart surgical repair requires cardiopulmonary bypass (CPB), as well as total sternotomy, which is physically and psychologically traumatic, particularly for paediatric patients [4]. Furthermore, percutaneous transcatheter device closure of the VSD can cause several adverse events, such as arrhythmia and device embolism, as well as vascular complications [5]. This patient's UCG showed a 5.9 mm residual shunt located under the pericardium patch and more than 2 mm from the tricuspid valve. According to the general condition and UCG of this patient, we created a new hybrid operative method to repair the residual shunt. With TEE guidance, we punctured the right ventricle with the 18 G needle directly through the right ventricular surface, and then we inserted the guide wire into the left ventricle through the sheath. Subsequently, the 8 mm closure device was released. This method helps to avoid the risk of open surgical repair and percutaneous transcatheter device closure. This method also offers a new avenue for the treatment of postoperative residual shunts in primary VSD and pulmonary valve replacement.

## 4. Conclusions

In summary, this was the first case of this type of surgery. Currently, we have performed three cases of this type of operation, and the patients have recovered very well. This method requires the surgeon to have extensive experience because selection of the puncture site must be very accurate during the operation. This method does not allow multiple choices of puncture sites, which would result in cardiac tamponade because of excessive bleeding. This surgery was attempted experimentally by our team, and our experience was far from sufficient. We hope to gain more experience and to conduct more extensive follow-up after surgery with the aim of providing a new, more secure, efficient, and reliable method of treatment for children with congenital heart disease.

## Abbreviations

CHDs: Congenital heart defects
VSDs: Ventricular septal defects
UCG: Ultrasonic cardiogram
ECG: Electrocardiography
TEE: Transesophageal echocardiograph.

## Additional Points

The design and performance of the current study involving human subjects were clearly described in a research protocol.
